# Expression of the paralogous tyrosine hydroxylase encoding genes *th1* and *th2* reveals the full complement of dopaminergic and noradrenergic neurons in zebrafish larval and juvenile brain

**DOI:** 10.1002/cne.22213

**Published:** 2009-09-16

**Authors:** Alida Filippi, Julia Mahler, Jörn Schweitzer, Wolfgang Driever

**Affiliations:** 1 Department of Developmental Biology, Institute of Biology I, University of FreiburgD-79104 Freiburg, Germany; 2 Freiburg Institute for Advanced Studies, School of Life SciencesD-79104 Freiburg, Germany

**Keywords:** catecholamines, dopamine, noradrenaline/norepinephrine, teleost CNS, zebrafish, genome duplication, paralogous genes, tyrosine hydroxylase

## Abstract

The development of dopaminergic and noradrenergic neurons has received much attention based on their modulatory effect on many behavioral circuits and their involvement in neurodegenerative diseases. The zebrafish (*Danio rerio*) has emerged as a new model organism with which to study development and function of catecholaminergic systems. Tyrosine hydroxylase is the entry enzyme into catecholamine biosynthesis and is frequently used as a marker for catecholaminergic neurons. A genome duplication at the base of teleost evolution resulted in two paralogous zebrafish tyrosine hydroxylase-encoding genes, *th1* and *th2*, the expression of which has been described previously only for *th1*. Here we investigate the expression of *th2* in the brain of embryonic and juvenile zebrafish. We optimized whole-mount in situ hybridization protocols to detect gene expression in the anatomical three-dimensional context of whole juvenile brains. To confirm whether *th2*-expressing cells may indeed use dopamine as a neurotransmitter, we also included expression of *dopamine beta hydroxylase*, *dopa decarboxylase*, and *dopamine transporter* in our analysis. Our data provide the first complete account of catecholaminergic neurons in the zebrafish embryonic and juvenile brain. We identified four major *th2*-expressing neuronal groups that likely use dopamine as transmitter in the zebrafish diencephalon, including neurons of the posterior preoptic nucleus, the paraventricular organ, and the nuclei of the lateral and posterior recesses in the caudal hypothalamus. *th2* Expression in the latter two groups resolves a previously reported discrepancy, in which strong dopamine but little tyrosine hydroxylase immunoreactivity had been detected in the caudal hypothalamus. Our data also confirm that there are no mesencephalic DA neurons in zebrafish. J. Comp. Neurol. 518:423–438, 2010. © 2009 Wiley-Liss, Inc.

Dopamine (DA) and noradrenaline (NA) are the major catecholamines (CA) in the central nervous system (CNS; Björklund and Dunnett,^2007^; Smeets and González,^2000^). DA systems contribute to control of motor activity, behavior, perception, and sleep and, at least in mammals, are involved in motivation, mood, reward, learning, and attention (Schultz,^2007^). NA plays an important role in the control of autonomic systems but also influences diverse processes in the brain, including attention or arousal and the reward system (Ordway et al.,^2007^). DA and NA system dysfunctions are involved in a variety of psychiatric and neurological disorders, such as Parkinson's disease and schizophrenia (Hirsch et al.,^1988^; Lang and Lozano,^1998^). The anatomical locations of CA neurons have been extensively described in amniotes (Smeets and González,^2000^), but, for the more recently emerging anamniote model organisms, which offer new experimental avenues for studying CNS development, not all aspects of CA system anatomy are understood.

For the mammalian catecholaminergic CNS neurons, a nomenclature is used in which CA neuronal clusters are basically numbered in a caudal-to-rostral order (Hökfelt et al.,^1984^). In mammals, noradrenergic groups are restricted to the hindbrain (caudal groups of medulla oblongata A1, A2, and rostral groups of the area of the locus coeruleus A6 as well as A4, A5, and A7). The major dopaminergic groups of mammals are in the retrorubral area (A8), substantia nigra pars compacta (A9), and ventral tegmental area (A10). Mesencephalic DA neurons have ascending projections to forebrain targets via the mesocortical, mesolimbic, and mesostriatal (or nigrostriatal) pathways (for review see Björklund and Dunnett,^2007^). Dopaminergic neurons are also located in the diencephalon (A11 to A15), olfactory bulb (A16), and inner nuclear layer of the retina (A17). The diencephalic DA groups correlate anatomically with A11, periventricular gray matter of thalamus, extending into midbrain; A12, tuberal cells of arcuate nucleus and adjacent periventricular nucleus; A13, zona incerta of ventral thalamus (also named prethalamus; Puelles and Rubenstein,^2003^); A14, rostral periventricular group; and A15, preoptic area/rostral hypothalamus (Smeets and González,^2000^).

Much of the classical work on catecholamine systems in fish has been performed by histochemical and immunohistological analysis of the adult brain, comprehensively reviewed for cartilagenous fish by Stuesse et al. (1994) and for bony fish by Meek (1994). With regard to teleost catecholamine system development, most work has focused on the stickleback (for review see Ekström et al.,^1994^) and more recently the zebrafish (*Danio rerio*). Zebrafish have a functional nervous system after 4–5 days of embryonic development, which allows complex behaviors such as swimming and hunting and thus provides an excellent model for studying vertebrate brain development. Well-established genetics, a transparent nervous system, and many experimental tools make the zebrafish an excellent system with which to study mechanisms of catecholaminergic system development. Noradrenergic cell groups are located in the locus coeruleus (LC), medulla oblongata (MO), and area postrema (AP; Ma,1994b,^1997^), as in mammals. However, teleost DA brain architecture differs from that of mammals. Most significantly, zebrafish have been reported to lack DA neurons in the mesencephalon (Holzschuh et al.,^2001^). Unlike the case for mammals, significant DA groups are also found in the subpallium (ventral telencephalon) and the pretectum. However, other tel- and diencephalic groups have correlates in amniotes, including DA cell groups in the olfactory bulb, retina, preoptic region, and ventral diencephalon, including the hypothalamus (Holzschuh et al.,^2001^; Kaslin and Panula,^2001^; Ma and Lopez,^2003^; McLean and Fetcho,^2004^; Rink and Wullimann,2002b). Based on anatomical location and cell shape, embryonic DA neurons in the diencephalon can be found in the ventral thalamus (group 1), posterior tuberculum (groups 2 and 4), and medial (group 3) and lateral hypothalamus (groups 5 and 6; Rink and Wullimann,2002b). Homology relationships from zebrafish to mammals have been established at the molecular level for some but not all zebrafish DA groups so far. The Otp homeodomain transcription factor-dependent zebrafish DA groups 2, 4, 5, and 6 correlate with the mouse A11 group, which also depends on Otp activity (Ryu et al.,^2007^).

A major enigma remains, the nature of teleost DA connectivity corresponding to ascending dimesencephalic DA projections from the substantia nigra and ventral tegmental area. A comparative analysis of the evolution of DA systems from teleosts to mammals suggests that mesencephalic DA neurons arose in the tetrapod lineage subsequent to caudal expansion or migration of ventral diencephalic DA cell groups, implying a close evolutionary relationship between these DA cell groups (Smeets and González,^2000^). In cartilaginous fishes, DA neurons have been detected in the midbrain, which could indicate that mesencephalic DA neurons might have independently evolved in cartilaginous fish or might have been secondarily lost in teleost (Meredith and Smeets,^1987^; for review see Stuesse et al.,^1994^). However, it so far has remained possible that neurons with DA activity in the teleost midbrain escaped detection as a result of divergence of DA marker genes. The possibility that zebrafish DA groups have remained undetected so far became more pressing when a second gene, *th2*, encoding tyrosine hydroxylase as entry enzyme of CA biosynthesis, was identified in the zebrafish genome (Candy and Collet,^2005^). Many zebrafish genes are present as two paralogs as a result of a genome duplication at the base of teleost evolution (Amores et al.,^1998^; Postlethwait et al.,^1998^, ^1999^). Candy and Collet (2005) focused their analysis on the teleost barramundi *Lates calcarifer*, for which they postulate, based on RT-PCR expression analysis and bioinformatic analysis of genome control regions, that both *th* genes may still be functional but differentially regulated at the transcriptional level.

To identify the full complement of dopaminergic and noradrenergic cell groups in the zebrafish CNS, here we analyze in detail the expression of both paralogous zebrafish *tyrosine hydroxylase* genes in the embryonic and the juvenile brain. Although the official nomenclature (www.ZFIN.org) currently distinguishes the well-studied *th* gene (Holzschuh et al.,^2001^) as well as the paralogous *th2* gene (Candy and Collet,^2005^), we will use the term *th1* (for *th*) to avoid confusion for the first published zebrafish gene as well as antisera derived for TH1 protein. We have also included *dopa decarboxylase* (*ddc*), *dopamine beta hydroxylase* (*dbh*), and *dopamine transporter* (*dat*) expression in our analysis, which, based on current genome information, all genes have only one homolog in zebrafish. Our data provide the first complete account of CA neurons in zebrafish embryonic and juvenile brain and confirm that indeed DA neurons do not develop in the zebrafish mesencephalon.

## MATERIALS AND METHODS

### Animals

Zebrafish (*Danio rerio*) embryos and larvae were bred and maintained under standard conditions (Westerfield,^2000^). For analyzing *th2* expression at embryonic stages, fertilized eggs obtained from AB/TL wild-type pairs were raised at 28°C and fixed in 4% paraformaldehyde in phosphate-buffered saline at the desired stages. For expression analyses in the juvenile brain, fish were raised until 30 days of development (10 mm stage), deeply anesthetized in 0.02% Tricaine (ethyl 3-aminobenzoate methanesulfonate; Sigma, St Louis, MO), and then decapitated. The brains were removed from the skull and immediately immersed in cold 4% paraformaldehyde. They were fixed at 4°C for 3 days, then dehydrated to 100% methanol and stored at –20°C until use. In total, 18 juvenile zebrafish brains were analyzed in the present study. All the experimental procedures were in accordance with the German laws for animal care, and a permit was obtained from Regierungspräsidium Freiburg to kill fish for organ removal and analysis.

### Whole-mount in situ hybridization

Standard colorimetric whole-mount in situ hybridization (WISH) was performed according to Hauptmann and Gerster (1994). The protocol was in most steps identical for both embryos and whole brains. The methanol-stored specimens were rehydrated to phosphate-buffered saline containing 0.1% Tween 20 (PBST) and permeabilized by proteinase K treatment (10 μg/ml; an incubation time of 10 minutes is sufficient for permeabilizing the 30-day-old brains). The prehybridization was carried out at 65°C in hybridization mix (HM: 50% formamide, 5× SSC, 50 μg/ml heparin, 5 mg/ml torula RNA, 0.1% Tween 20), and hybridization in HM with the specific digoxigenin-labeled antisense probe. After formamide/SSC and PBST washing series (Hauptmann and Gerster,^1994^), the specimens were blocked with 2% sheep serum and 2 mg/ml bovine serum albumin (BSA) in PBST and incubated overnight with anti-digoxigenin alkaline phosphatase-conjugated antibodies (1:3,000; Roche) at 4°C. After PBST washes, the specimens were equilibrated in NTMT buffer (100 mM Tris-HCl, pH 9.5, 100 mM NaCl, 50 mM MgCl_2_, 0.1% Tween 20) and then incubated in NTMT staining buffer plus 450 μg/ml nitro-blue tetrazolium chloride (NBT) and 170 μg/ml 5-bromo-4-chloro-3′-indolyphosphate p-toluidine salt (BCIP). Once the staining reaction was completed, the specimens were extensively washed with PBST and stored at 4°C in stop solution (PBST; 1 mM EDTA, pH 8) until further processing.

For WISH, the following antisense digoxigenin-labeled riboprobes were generated: *th1*: nucleotides 272–1092 of GenBank accession No. NM_131149 (named *th* in Holzschuh et al.,^2001^); *slc6a3/dat*: nucleotides 376–1055 of GenBank accession No. NM_131755 (Holzschuh et al.,^2001^); *dbh*: nucleotides 566–1351 of GenBank accession No. NM_001109694 (nucleotides 506–1291 of the coding sequence; Holzschuh et al.,^2003^); *ddc*: nucleotides 1063–2377 of GenBank accession No. NM_213342).

A zebrafish *th2* fragment was amplified by RT-PCR from 24-hours-postfertilization (hpf) embryos RNA using the following primers: *th2*-F 5′-CAGCTTCGTGTTTGAGGAGGAG-3′; *th2*-R 5′-CCTCAAAGCTCTCGGACACAAAGT-3′. These primers amplify a fragment comprising nucleotides 191–1300 of GenBank accession No. NM_001001829. The derived fragment was cloned into pCRII-TOPO (Invitrogen, LaJolla, CA), verified by sequencing, and used for antisense riboprobe generation.

### Fluorescent WISH and immunohistochemistry

Tyramide signal amplification (TSA)-based fluorescent in situ hybridization on whole-mount brains was performed as previously described for embryos (Filippi et al.,^2007^). The procedure was essentially the same as that for embryo WISH, with a few modifications. 1) On the first day, prior to proteinase K treatment, the brains were incubated in 1% H_2_O_2_ in PBST for 30 minutes to quench endogenous peroxidase activity. 2) On the second day, for the last wash after the hybridization step, we used TNT buffer (100 mM Tris-HCl, pH 7.5, 150 mM NaCl, 0.5% Tween 20) instead of PBST; the brains were then blocked with 1% blocking reagent (Roche; No. 1096176) in TNT buffer and incubated overnight with a 1:400 dilution of a peroxidase-conjugated anti-digoxigenin antibody (Roche; No. 1207733) to detect the probe. 3) On the third day, the brains were washed extensively with TNT buffer and incubated for 1 hour in the dark with the tyramide-Alexa488 working solution (Invitrogen-Molecular Probes; TSA kit No. 12), prepared according to the kit instructions.

After F-WISH, the brains were washed, equilibrated with PBST containing 1% DMSO (PBTD), and preincubated for at least 2 hours with blocking solution (10% goat serum and 1% protease-free BSA in PBTD). They were then incubated overnight with a 1:500 rabbit polyclonal anti-TH1 primary antibody (Ryu et al.,^2007^), subsequently detected by a 1:1,000 Alexa555-conjugated secondary goat anti-rabbit antibody (Invitrogen-Molecular Probes). Although the antibody was designated anti-TH by Ryu et al. (2007), we will use the name *anti-TH1* to distinguish clearly the proteins derived from the two paralogous genes.

### Characterization of TH1 antibody

**Figure 1:**
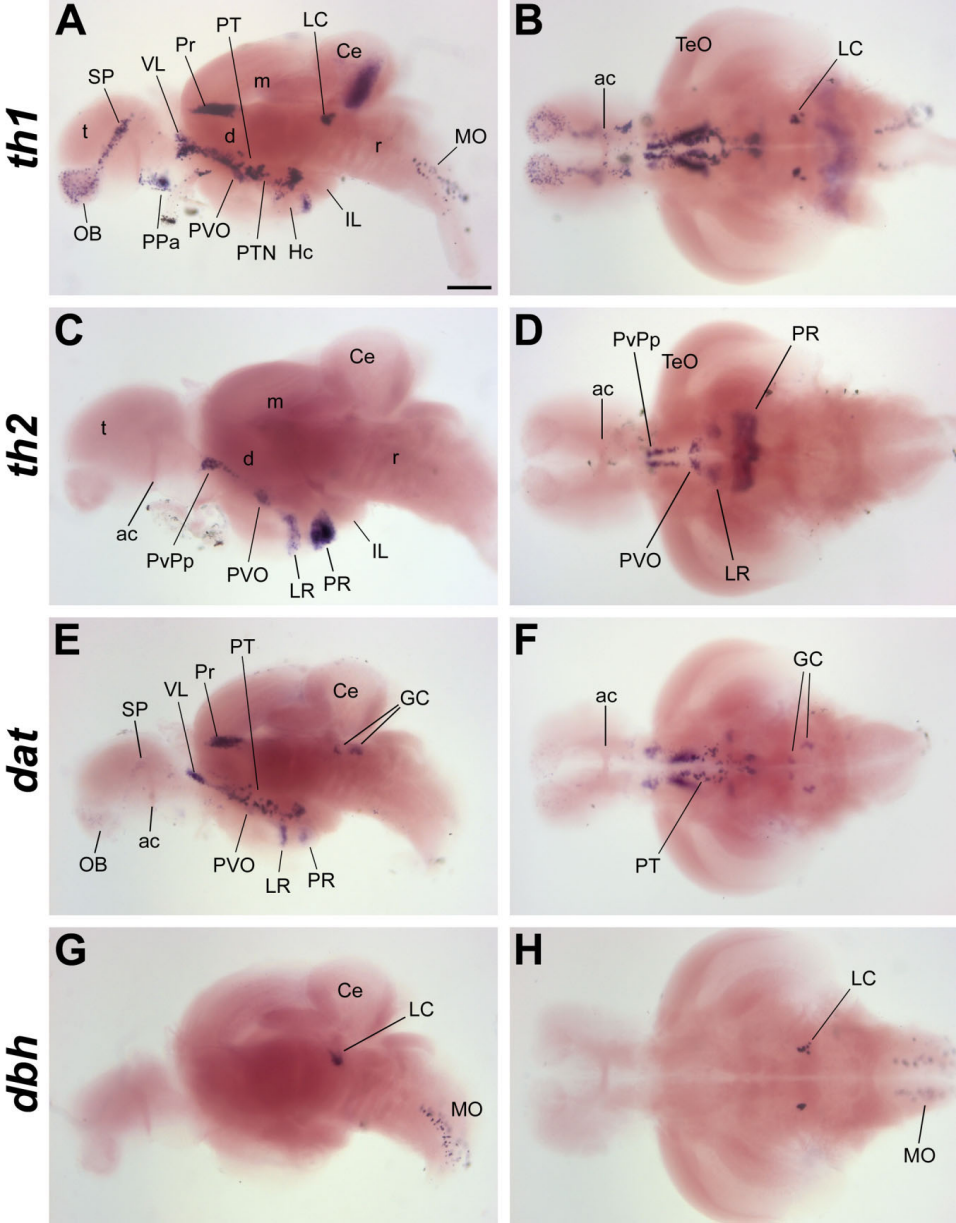
Expression of *th1* and *th2* as well as dopaminergic and noradrenergic markers in the brain of 1-month-old zebrafish. *th1* (**A,B**), *th2* (**C,D**), *slc6a3/dat* (**E,F**), And *dbh* (**G,H**) expression in 30-day-old zebrafish brains was detected by WISH. A,C,E,G are lateral views, dorsal at top, and B,D,F,H are dorsal views, anterior to the left. For the dorsal views, the images focus on an intermediate plane at the level of the posterior tuberculum for *th1*, *th2* and *dat* and at the level of the locus coeruleus for *dbh*. For abbreviations see list. Scale bar = 100 μm.

**Figure 2:**
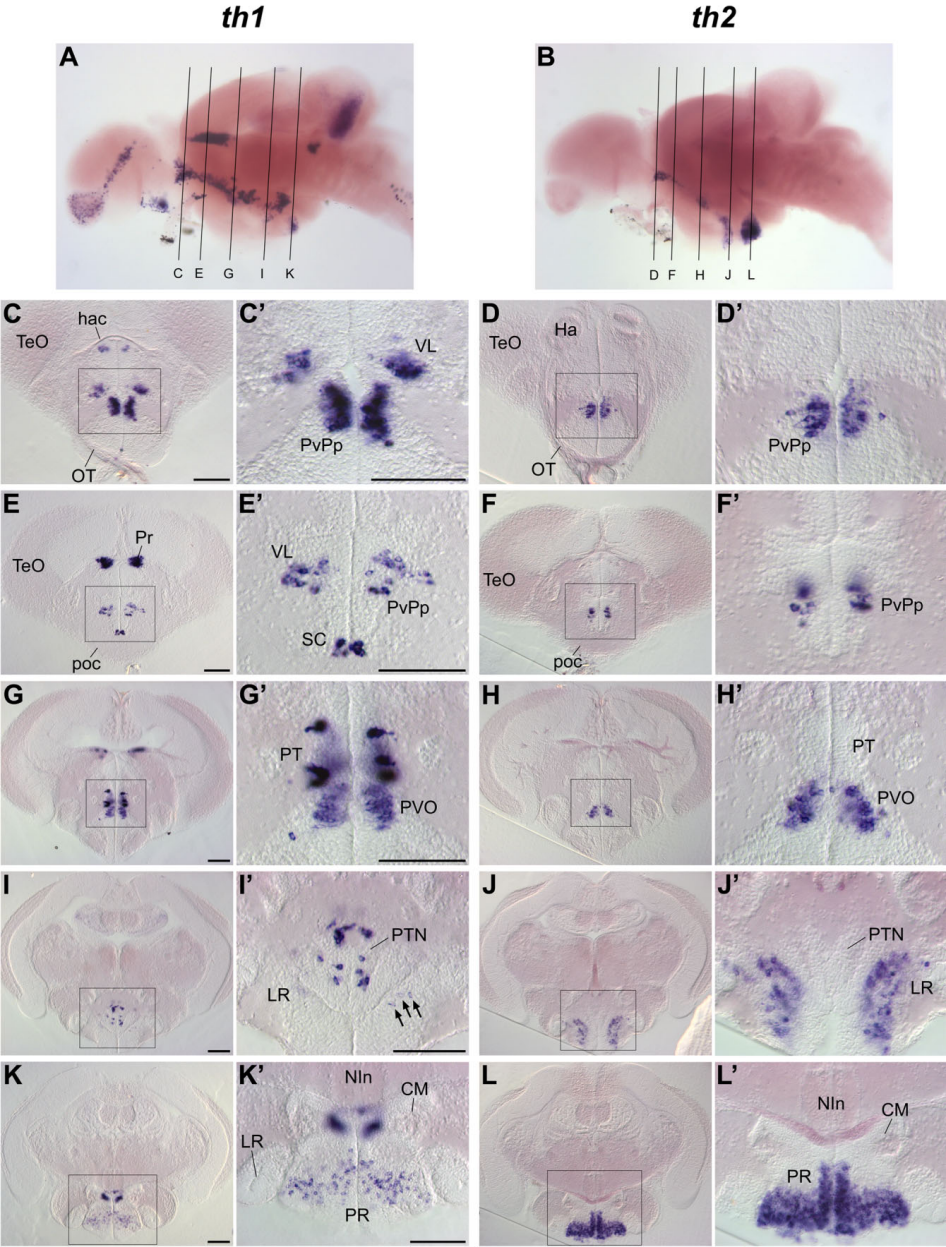
Comparison of *th1* and *th2* expression in the brain of 1-month-old zebrafish. *th1* And *th2* expression in 30-day-old zebrafish brains was detected by WISH. **A,B:** Lateral overviews of whole brains stained for *th1* (A) and *th2* (B) as in Figure 1A,C. C–L: Transverse sections showing *th1* (**C,E,G,I,K**) and *th2* (**D,F,H,J,L**) distribution in the diencephalic areas indicated by the vertical lines in A and B, respectively. **C′–L′** are higher magnifications of the framed areas in C–L. For abbreviations see list. Scale bars = 100 μm.

The anti-TH1 antibody had been generated as follows: a 824-bp fragment corresponding to nucleotides 274–1097 of the zebrafish *th1* gene (GenBank accession No. NM_131149) was PCR amplified and cloned into the pTRC-hisB vector (Invitrogen). This part of the protein includes amino acids 92–365 (see Supp. Info. Fig. 1). Protein was overexpressed in *Escherichia coli* according to standard procedures and purified using a Ni+NTA column (Qiagen, Germany) under denaturing conditions. The production of the rabbit antiserum was performed by Sigma Genosys (Cambridge, United Kingdom). Terminal bleed of one immunized rabbit (number 2914) was used in all experiments. The specificity of the serum was initially tested by using a Western blot. The serum, at 1:200 dilution, recognized 2 ng of the recombinant TH1 protein as a specific band on the Western blot. For whole-mount immunohistochemistry, we further processed the antiserum by extensive preabsorption against fixed 18–24-hpf zebrafish embryos, stages at which no or very little TH1 is expressed (50 μl of serum preabsorbed against about 500 embryos in blocking solution). To demonstrate the specificity of the antibody, we performed F-WISH to *th1* combined with anti-TH1 immunohistochemistry as described above. We observed TH1 immunoreactivity in all *th1*-expressing cell somata as well as in axons projecting from these cells (Supp. Info. Fig. 2), whereas omission of the primary antibody abolished the specific signal. The TH1 antibody did not stain any cell somata in the brain that did not express *th1*.

### Vibratome sections

Stained brains were embedded in 3% agarose/PBS and sectioned using a Leica VT1000S vibratome. Brains stained with the standard colorimetric reaction were cut in 30-μm slices, whereas those fluorescently labeled were cut in 80-μm slices for confocal imaging. All the slices were mounted in Aqua-Poly/Mount (Polyscience, Warrington, PA).

### Microscopy and imaging

Embryos and whole brains were mounted in 80% glycerol for microscopic observation. Light images were acquired with a Zeiss Axioskop2 compound microscope equipped with a Zeiss AxioCam MRc digital camera. Confocal z-stacks of the fluorescently labeled brain sections were recorded with a Zeiss LSM 5 Duo laser scanning confocal microscope. Z-projections from subsets of focal planes were generated with the Zeiss LSM software and exported as image-format files. The images were assembled into figures and processed in Adobe Photoshop CS2 9.0. Slight adjustments of contrast and brightness were made by using the Photoshop Levels or Brightness/Contrast functions.

## RESULTS

### Expression of *th2* in relation to *th1* and other catecholaminergic markers in the juvenile zebrafish brain

The identification of a second *tyrosine hydroxylase* gene, *th2*, in zebrafish and other teleosts (Candy and Collet,^2005^) opened up the possibility that the cell groups previously reported to express *th1* (Guo et al.,^1999^; Holzschuh et al.,^2001^) might not represent the full complement of catecholaminergic neurons in the zebrafish brain. Here, we investigated the expression of both *th* paralogous genes in relation to other catecholaminergic markers. Previous analysis of zebrafish tyrosine hydroxylase expression has utilized in situ hybridization (Holzschuh et al.,^2001^) or immunohistochemistry with antibodies against mammalian TH (Kaslin and Panula,^2001^; Ma,1994a; Rink and Wullimann,2002b) or zebrafish-specific TH1 antibodies (Ryu et al.,^2007^). Zebrafish TH2, despite its sequence similarity to TH1, is significantly more diverged from human TH than TH1 (Supp. Info. Fig. 1). We found, based on our experience with both zebrafish anti-TH1 (see also Fig. 4) and commercial mammalian anti-TH antibodies, that they may not efficiently detect zebrafish TH2. In fact, when comparing *th1* expression and anti-TH1 immunohistochemistry in the adult zebrafish brain, we could demonstrate that our TH1 antibody specifically detects *th1*-expressing catecholaminergic neurons (Supp. Info. Fig. 2). Therefore, we focused our analysis of *th2* expression on detection of *th2* mRNA by in situ hybridization.

To compare expression domains best in the context of the intact anatomy of the brain, we wanted to perform expression analysis with both whole intact brain preparations and histological sections. For this analysis, we considered a stage of development when the brain morphology resembles that of the adult zebrafish brain and is large enough to be easily dissected but, at the same time, is still small enough to allow analysis of gene expression by WISH. For these reasons, we decided to analyze the brains of 30-day-old juvenile zebrafish. We found that at this stage of growth the brains are still readily permeabilized and accessible to riboprobes and antibodies, offering us the possibility to visualize how the transcripts of enzymes involved in catecholamine biosynthesis and dopamine transporter proteins are distributed (Fig. 1A–H).

Previously, WISH analysis of whole juvenile brains has not been characterized in detail. Analysis of the expression of *th1* in the brains of 30-day-old juvenile zebrafish served as proof-of-principle that WISH at this size of brain growth is able to detect efficiently specific messages in all domains of the brain. In fact, all previously reported CA cell groups (Kaslin and Panula,^2001^; Ma,^2003^; Rink and Wullimann,2002b) could be detected by WISH (Fig. 1A,B): DA groups in olfactory bulb, subpallium, preoptic region, ventral thalamus, posterior tuberculum, paraventricular organ, posterior tuberal nucleus, dorsomedial and caudal hypothalamus, and pretectum. NA neurons were detected in the locus coeruleus and the medulla oblongata.

In contrast to the case for *th1*, we found *th2* expression confined to the diencephalon, and no transcripts were detected in telencephalon or rhombencephalon (Fig. 1C,D). In addition, given that there have been disputes about whether there are mesencephalic DA neurons in zebrafish, our data clearly show that there are no *th2*-expressing cells in the mesencephalon. Within the diencephalon, *th2*-expressing cells were restricted to the basal plate and appeared to be concentrated in four major hypothalamic groups, including the posterior part of the preoptic nucleus, the paraventricular organ, and two groups in the caudal hypothalamus.

Expression of *dopamine transporter* (*slc6a3/dat*), which mediates the reuptake of dopamine, identifies dopaminergic neurons that regulate synaptic transmission by control of dopamine in the synaptic cleft. *dat* Expression is not observed in NA neurons and can thus help to distinguish DA from NA neurons. NA neurons can be positively defined by the expression of *dopamine beta hydroxylase* (*dbh*). In the whole brain, the expression of *slc6a3/dat* mRNA resembled that of *th1*; however, the staining intensity, which likely correlates with expression levels, was very weak for *dat* in some regions, including the olfactory bulb, the subpallium, and the preoptic region (Fig. 1E,F). However, detailed analysis at higher magnification revealed that *dat* expression could also be detected in those regions (data not shown). As already observed in the embryonic brain (Holzschuh et al.,^2001^), *slc6a3/dat* was also expressed in the hindbrain in the area of the griseum centrale, in two groups of cells that did not express either *th1* or *th2* (Fig. 1E,F). Compared with the noradrenergic neurons of the locus coeruleus, as labeled by *dbh* expression (Fig. 1G,H), one of the rhombencephalic *slc6a3/dat*-expressing cell groups appeared to be located medial and the second slightly caudal to the noradrenergic LC neurons (compare Fig. 1F with H). *dbh* Itself in the CNS was expressed exclusively in the locus coeruleus and medulla oblongata of the hindbrain.

### Distribution of *th2* transcripts in the juvenile brain and comparison with *th1* expression

To determine the exact neuroanatomical positions of *th2*-expressing neurons, we performed transversal sections of the whole-mount-stained brains (Fig. 2C–L′). As reference both for structure and for nomenclature, we used the atlas of the adult zebrafish brain (Wullimann et al.,^1996^).

In contrast to *th1*, no *th2* transcripts were detected in the ventrolateral thalamic nucleus (VL; Fig. 2, compare C′ with D′). The rostralmost *th2*-expressing cell group was located in the posterior preoptic nucleus. It consisted of an anterior dense domain with a ventrocaudal extension, to which fewer cells contributed (Fig. 2B,D,D′,F,F′). The posterior part of this group was clearly located in the area identified by Wullimann et al. (1996) as the posterior part of the parvocellular preoptic nucleus (PPp; Fig. 2F,F′). Conversely, at least a part of the predominant anterior portion appeared to be in the area of the magnocellular preoptic nucleus (PM; Wullimann et al.,^1996^; Fig. 2D,D′). To avoid confusion, we will use the more general term of *posterior periventricular preoptic nucleus* (PvPp) to identify these potentially heterogeneous populations of *th2*-expressing cells, without discriminating between their magno- and parvocellular components. *th1* And *th2* were both expressed in the PvPp, in apparently overlapping domains (compare Fig. 2C,C′ with D,D′ and E,E′ with F,F′). Some of the posterior *th2*-expressing cells appeared to be located slightly ventral to the *th1*-expressing neurons of the same area (Fig. 2E–F′) and thus closer to the suprachiasmatic nucleus (SC), which contained only *th1*-positive cells (Fig. 2E′,F′). In the dorsal diencephalon, a massive domain of *th1* expression was detected in the pretectum, completely devoid of *th2* transcripts (compare Fig. 2E with F).

A second cluster of *th2*-expressing cells was detected in the paraventricular organ (PVO; Fig. 2H,H′). In this region, *th1* is expressed in a numerous population of bipolar liquor-contacting neurons, just ventral to the few large, multipolar, pear-shaped neurons of the posterior tuberculum (PT; Ma,^2003^; Rink and Wullimann,2002b). These two cell types were clearly distinguishable by size and *th1* transcript expression level (Fig. 2G,G′). The number, morphology, and staining intensity of *th1*- and *th2*-expressing cells in the PVO were very similar (compare Fig. 2G′ with H′), suggesting that this neuronal group possibly coexpresses both genes. The overlap was restricted to the PVO, insofar as no expression of *th2* was observed in the posterior tubercular neurons (Fig. 2H′).

Caudal to the PVO and ventral to the posterior part of the PT extends the posterior tuberal nucleus (PTN), which contained *th1*-expressing neurons in its most caudal-ventral part (Fig. 2I,I′) but no *th2*-positive cells (Fig. 2J,J′). Farther ventral to it, and extending caudally until the posterior recess of the diencephalic ventricle, lie the regions anatomically defined as the dorsal and caudal zones of the periventricular hypothalamus (Hd and Hc, respectively; Wullimann et al.,^1996^), which comprise the nuclei surrounding the lateral and posterior recesses of the diencephalic ventricle. We found in the anteriormost part of this region a third fairly large group of *th2*-expressing neurons (Fig. 2J,J′), whereas only very few and weakly stained *th1*-positive cells could be detected (arrows in Fig. 2I′). This area corresponds to the nucleus of the lateral recess (LR), which has been shown to contain intense DA immunoreactivity but few or no TH-ir cells in other teleosts (Batten et al.,^1993^; Ekström et al.,^1990^, ^1992^; Meek and Joosten,^1993^; Meek et al.,^1989^; Sas et al.,^1990^; Yoshida et al.,^1983^).

Finally, the fourth and largest cluster of *th2*-expressing cells was found in the nucleus of the posterior recess (PR; Fig. 2L,L′). The rostralmost part of this nucleus appeared to express exclusively *th2* (not shown). However, in its posterior subregion, *th2* overlapped with *th1* expression (compare Fig. 2K,K′ with L,L′), which was, however, detectable in a smaller number of cells only in this area. This cluster of DA neurons forms a population of liquor-contacting cells around the posterior recess of the diencephalic ventricle (Ma,^2003^; Rink and Wullimann,2002b).

**Figure 3:**
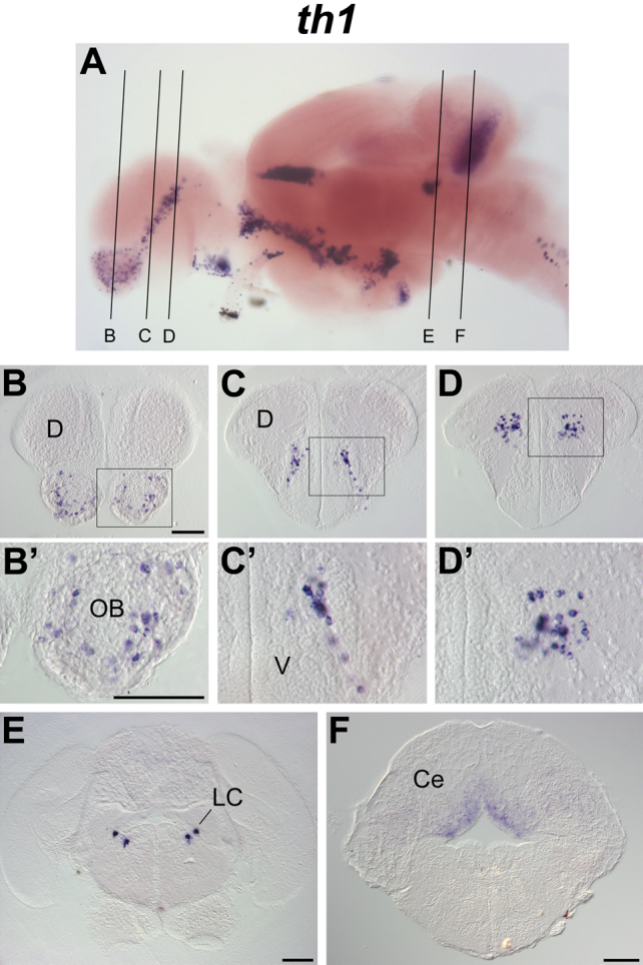
*th1* Expression in telencephalon and hindbrain of 1-month-old zebrafish. **A:** Lateral overview of *th1* expression in the whole brain as in Figure 1A. **B–F:** Transverse sections through the olfactory bulb (B), the telencephalon (C,D), the locus coeruleus (E), and the cerebellum (F). For abbreviations see list. Scale bars = 100 μm in B (applies to B–D); 100 μm in B′ (applies to B′–D′); 100 μm in E,F.

Given that *th1* expression detection by WISH has previously not been reported for juvenile or adult brains, we completed our analysis of *th1* expression by analyzing histological sections also for those brain territories in which *th2* is not expressed (Fig. 3). *th1*-Expressing neurons were broadly found in the olfactory bulb, where they appeared to be located mostly in, or adjacent to, the glomerular layer (Fig. 3B) and thus likely correspond to the periglomerular and/or mitral DA neurons previously described for zebrafish (Byrd and Brunjes,^1995^). Additional *th1*-expressing neurons were located in the subpallium, at the border between the dorsal and ventral subdivisions of the telencephalic area (Fig. 3C,D). NA neurons expressing *th1* were detected in the locus coeruleus of the hindbrain (Fig. 3E) and the medulla oblongata (no transversal sections shown). In addition, a *th1* signal was present in the cerebellum (Fig. 3F), but it was of a diffuse nature and may represent *th1* transcripts transported there by catecholaminergic projections that innervate the cerebellum. Transport of *tyrosine hydroxylase* mRNA has previously been reported for mammalian DA neurons (Skutella et al.,^1994^). Although the corpus cerebellaris has been reported to be poorly innervated by catecholaminergic projections (Kaslin and Panula,^2001^; Ma,^1997^), another study found a dense catecholaminergic plexus at rostral levels of the corpus cerebelli in a gymnotiform fish (Sas et al.,^1990^). Therefore, the *th1*-positive signal might not be explained simply by high density of CA projections. Given that we did not observe either *ddc* or *slc6a3/dat* expression in the same area, we conclude that there are no CA neurons in the zebrafish cerebellum.

### Distinct and overlapping domains of *th2* expression and TH1 immunoreactivity

**Figure 4:**
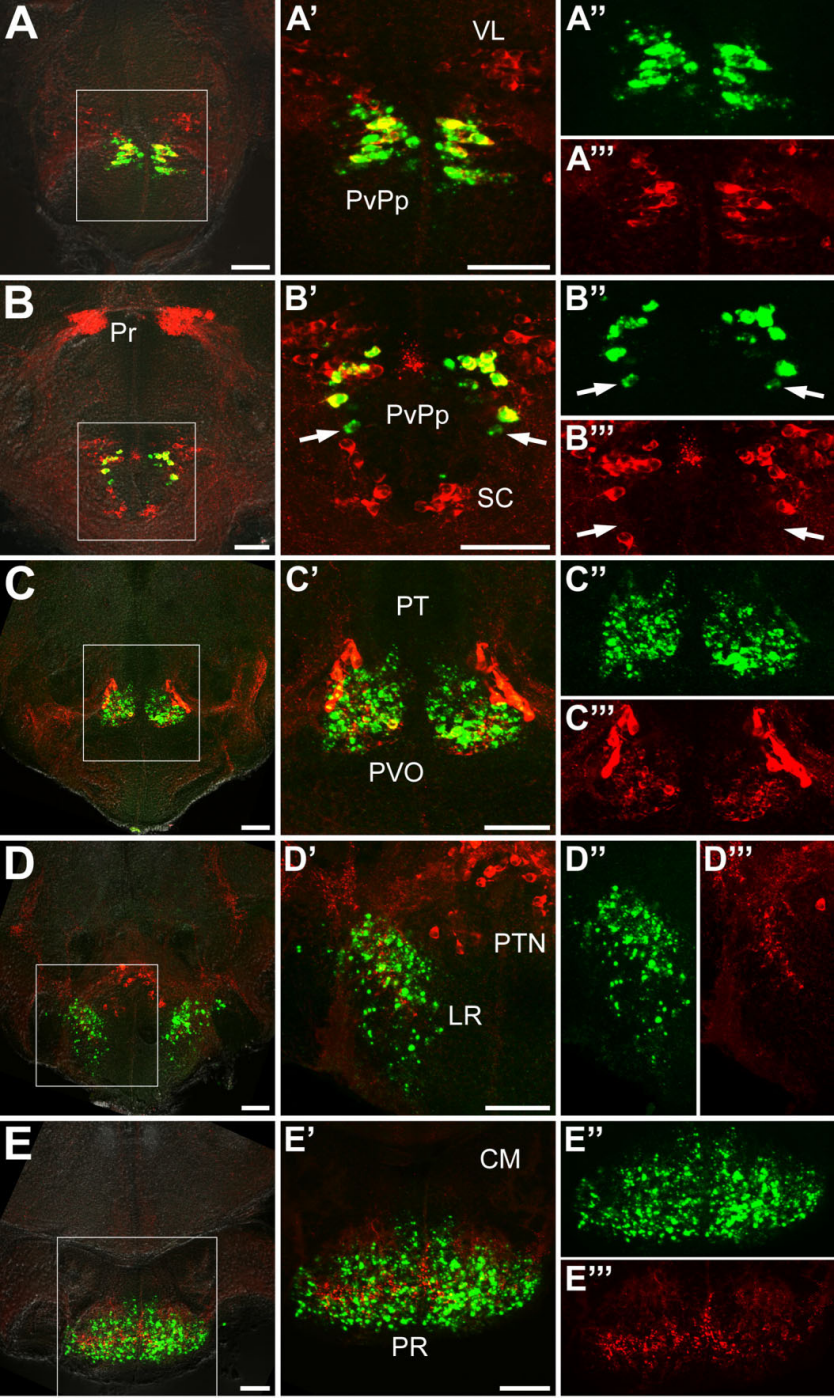
Localization of *th2*-expressing cells and TH1-immunoreactive neurons in the diencephalon. F-WISH to detect *th2* expression (green) coupled with anti-TH1 immunofluorescence (red). A–E: Transverse sections through the equivalent *th2*-positive territories displayed in Figure 2. The images are 7–14-μm confocal z-projections of areas comprising *th2*-expressing cells. For each projection, we present an overview (**A–E**) and a higher magnification of the framed areas (**A′–E′**).**A′′–E′′,A′′′–E′′′** show the individual channels. Most *th2*-expressing cells are TH1-ir in the posterior periventricular preoptic nucleus (A–B′′′) and in the liquor-contacting neurons of the paraventricular organ (C–C′′′), although cells exclusively expressing *th2* can be detected too (arrows in B′–B′′′). In contrast, almost exclusive domains of *th2* expression are found in the nuclei of the lateral (D–D′′′) and posterior (E–E′′′) recesses, where the TH1-ir contribution is minor. A magenta-green copy of this figure is available as Supporting Information Figure 3. For abbreviations see list. Scale bars = 50 μm.

The results of our expression analysis suggest that *th2* may be coexpressed with *th1* in some areas of the diencephalon but also has its distinct expression domains. To clarify further the spatial relationship between *th1*- and *th2*-expressing cells, we fluorescently labeled the *th2* transcripts by whole-mount F-WISH and detected TH1 protein by immunohistochemistry with a specific anti-TH1 antibody (Ryu et al.,^2007^). We then made transverse sections to visualize better the stained areas by confocal microscopy. The results are presented in Figure 4; the transverse planes were chosen to correspond with those in Figure 2 (compare Fig. 4A–E with Fig. 2C,D, E,F, G,H, I,J, and K,L, respectively).

In the posterior part of the periventricular preoptic nucleus, most of the rostral *th2*-expressing neurons (green) were also TH1-ir (red, Fig. 4A–A′′′). In the caudal extension of this area, there was a high degree of overlap too, but some neurons clearly expressing only *th2* were found here (Fig. 4B–B′′′, arrows).

In the paraventricular organ, most TH1-ir cells appeared to express *th2*, whose domain was apparently larger (Fig. 4C–C′′′). However, because *th2*-expressing cells appeared packed and intensely stained, it was difficult to discriminate between single- and double-labeled neurons.

As predicted by the comparison of *th1* and *th2* single WISH, the nucleus of the lateral recess had a predominant contribution of *th2*-expressing cells, whereas only few TH1-ir neurons were detected (Fig. 4D–D′′′). Similarly, the nucleus of the posterior recess was densely populated with *th2*-expressing cells, and only a fraction of those, the caudalmost ones, were also TH1-ir (Fig. 4E–E′′′).

Altogether, our data show that zebrafish *th2* expression is confined to the diencephalon in some domains predominantly overlapping those of *th1* expression, such as the preoptic area and the paraventricular organ, as well as in areas containing no or little *th1* contribution, such as the nuclei of the lateral and posterior recesses.

### Most *th2*-expressing cells likely correspond to neurons using dopamine as neurotransmitter

Next we asked whether *th2*-expressing cells may use dopamine as neurotransmitter. Dopaminergic neurons have to express *ddc* for neurotransmitter synthesis and *slc6a3/dat* for reuptake of DA from synaptic cleft, if the principle mode of action is synaptic and not neurosecretory. Therefore, we made transverse sections of brains hybridized with either *slc6a3/dat* or *ddc* riboprobes and analyzed their expression with particular attention to the anatomical areas where we detected *th2* transcripts.

**Figure 5:**
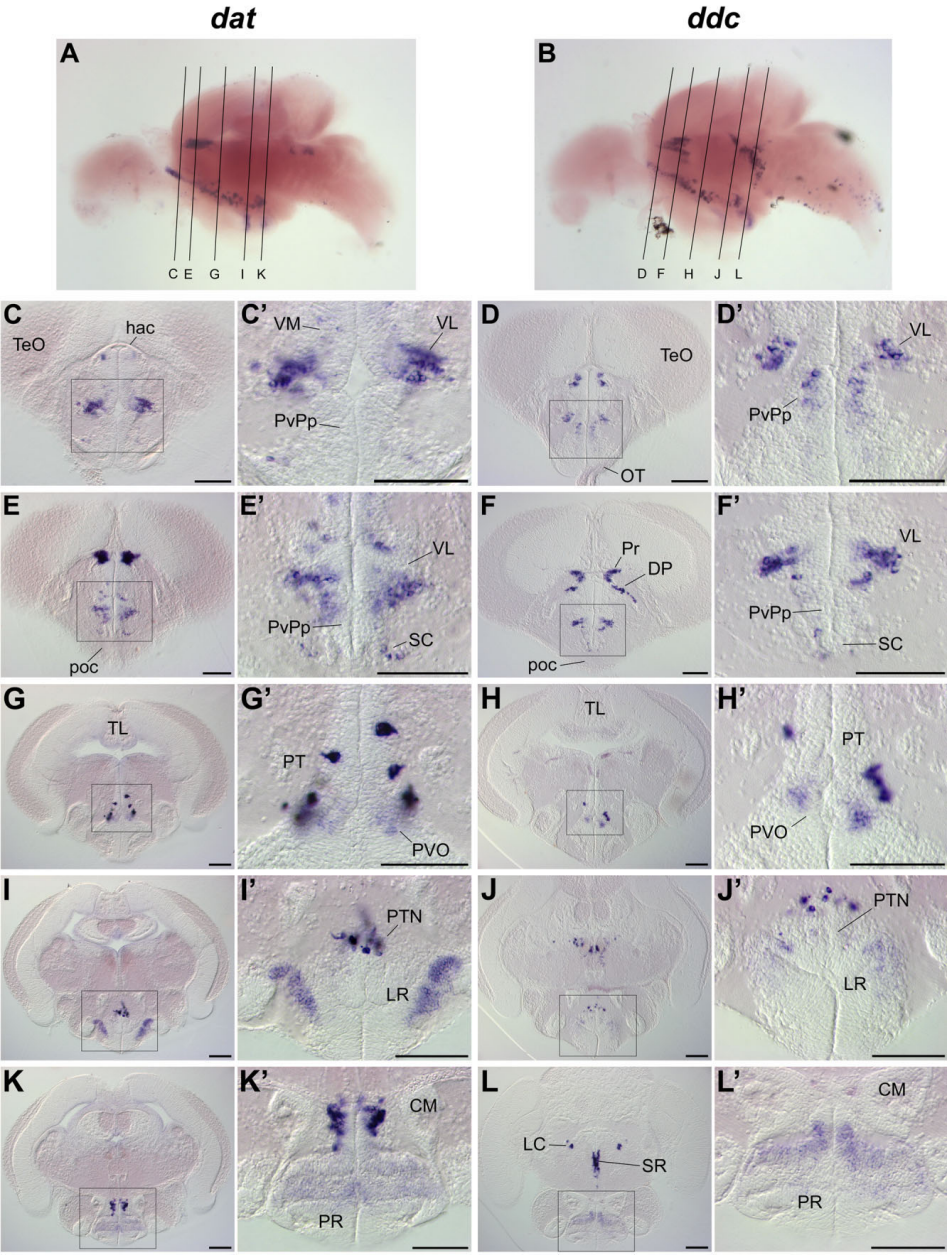
*slc6a3/dat* And *ddc* expression in *th2*-expressing areas indicates that *th2*-expressing cells are dopaminergic. **A,B:** Lateral overviews of whole brains stained for expression of *slc6a3/dat* (A; same as Fig. 1E) and *ddc* (B). **C–L:** Transverse sections at the levels indicated by the vertical lines in A and B show *slc6a3/dat* (C,E,G,I,K) and *ddc* (D,F,H,J,L) distribution in the same diencephalic areas that express *th2* (compare with Fig. 2). **C′–L′** are high magnifications of the areas boxed in C–L. For abbreviations see list. Scale bars = 100 μm.

For the rostral portion of PvPp, we did detect *ddc* (Fig. 5D,D′), and thus cells likely synthesize dopamine. However, we did not detect significant amounts of *slc6a3/dat* transcripts (Fig. 5C,C′). A faint signal was observed in a few cells, indicating that *slc6a3/dat* expression in this nucleus is possibly so weak that it is hardly detectable by our in situ hybridization protocol. However, because we consistently detected cells also in serial sections from other brains (data not shown), we assume that *dat* is likely expressed in some or all of the *th2*-expressing cells. A strong signal for both *dat* and *ddc* was instead present in the ventrolateral thalamic nucleus, and we additionally observed *dat* expression in more dorsal scattered cells of the ventromedial thalamic area, which by anatomical comparison of section planes were *th1*-negative.

In the caudal portion of PvPp, *slc6a3/dat*-expressing cells could be detected in a rather ventral position, suggesting that the *th2*-positive cells (more ventrally located compared to *th1*-expressing neurons) may express *slc6a3/dat* in this area (Fig. 5E,E′, compare with Fig. 2E′,F′). These neurons were located in the same area where we detected *ddc* expression (Fig 5F,F′), indicating that *th2*-expressing cells in this area may express *dat* and *ddc* as well.

*slc6a3/dat* Was expressed also in the PVO, although faintly (Fig. 5G,G′), as well as in the LR (Fig. 5I,I′). This latter region was almost devoid of *th1*-expressing neurons (see Fig. 2I′), so the numerous *slc6a3/dat*-positive cells found here likely represent dopaminergic neurons expressing *th2* (compare with Fig. 2J′). Both territories also expressed *ddc*, although expression levels in the LR were low (Fig. 5H,H′,J,J′). Finally, the neurons lining the posterior recess, in the caudalmost part of Hc, expressed both *slc6a3/dat* (Fig. 5K,K′) and *ddc* (Fig. 5L,L′), but also in this case *dat* expression was very weak. In summary, *dat* and *ddc* expression data indicate that most *th2*-expressing cells may indeed function as dopaminergic neurons.

### Embryonic expression of *th2*

To determine when the earliest *th2*-expressing neurons arise during development, we performed WISH on zebrafish embryos at embryonic and early larval stages up to 5 days of development. In our hands, *th2* transcripts were extremely difficult to detect, even though probe length, complexity, and concentration were similar to those of *th1*. Accordingly, the incubation time chosen for developing the colorimetric stain was much longer for *th2* than for *th1*. We interpret this as reflecting the fact that *th2* is probably very weakly expressed at embryonic stages.

**Figure 6:**
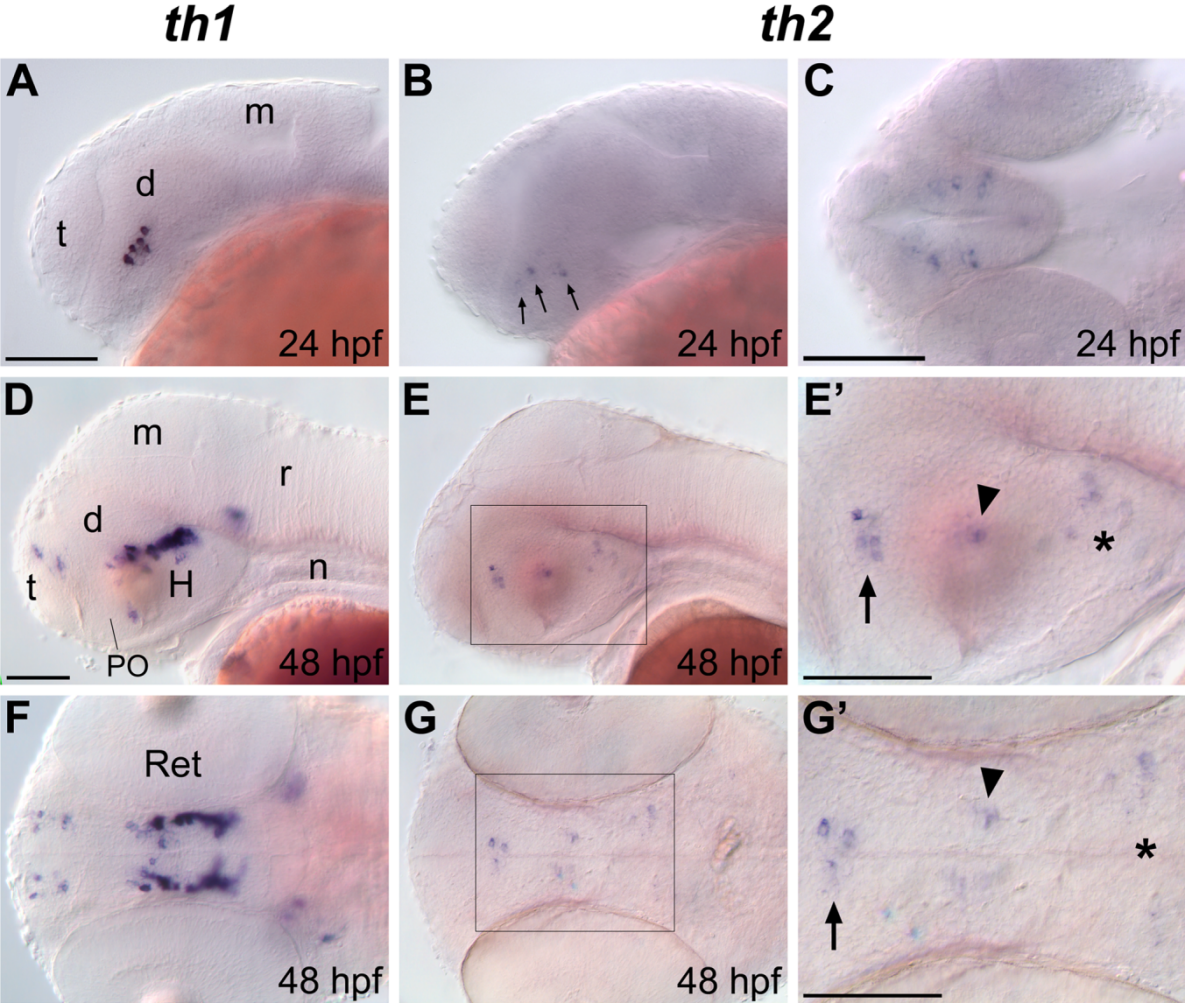
*th2* Expression at embryonic stages. WISH to detect *th1* (**A,D,F**) and *th2* (**B,C,E,E′,G,G′**) expression in embryos at 24 (A–C) and 48 (D–G′) hpf. Lateral (A,B,D,E; dorsal up) and dorsal (C,F,G) views of the head are presented, anterior to the left. E′ and G′ are high magnifications of the boxed areas in E and G, respectively. The developing *th2*-expressing nuclei in the preoptic area (arrow), paraventricular organ (arrowhead), and caudal hypothalamus (asterisk) are indicated in E′ and G′. For abbreviations see list. Scale bars = 100 μm in A (applies to A,B); 100 μm in C; 100 μm in D (applies to D–G); 100 μm in E′,G′.

Figure 6 shows the *th2* expression pattern at 24 and 48 hpf and compares it with that of *th1* expression. We could detect the earliest *th2*-expressing neurons in the ventral diencephalon already at 24 hpf, located anteroventral to the first *th1*-positive cells (Fig. 6, compare B with A). This region corresponds to the preoptic area and hypothalamus.

At 48 hpf, when the anatomical subdivisions are easier to discern, *th2*-expressing neurons appeared grouped in three main clusters (Fig. 6E,E′,G,G′). 1) A group was located in the periventricular preoptic area, just anterior to the developing dopaminergic neurons of the ventral thalamus labeled by *th1* expression (group 1, according to the numeric nomenclature proposed by Rink and Wullimann,2002b; Fig. 6, compare D with E and E′, and F with G and G′, arrow). This group likely corresponds to the future neurons of the PvPp. 2) A group was located in the intermediate hypothalamus, in the area populated by the liquor-contacting *th1*-expressing group 3 cells (Fig. 6F,G,G′ arrowhead), likely corresponding to the developing PVO. 3) A group was found in the posterior hypothalamus, which may represent the earliest neurons of the Hc (LR and/or PR; Fig. 6E,E′,G,G′, asterisk).

No *th2* expression was detected in any other brain subdivision, consistently with the restricted diencephalic distribution that we observed in the juvenile brain. At early larval stages, the number of *th2*-positive neurons appeared to increase slightly in the posterior hypothalamus, but the expression level remained low (data not shown). From these data, we conclude that *th2* plays a minor role in dopamine synthesis during early development and that it might be active predominantly in later-differentiating dopaminergic neurons in the juvenile and adult brain.

## DISCUSSION

A prerequisite for understanding neuronal circuits is the knowledge of the anatomical location and transmitter phenotype of its cellular components. Catecholaminergic neurons modulate activity of other neurons in local as well as far-ranging circuits, and it is thus implicit that all CA groups be identified before studies of anatomy and function of circuits may be initiated. Identification of all neurons of one neurotransmitter phenotype is complicated in zebrafish, because a genome duplication at the base of teleost evolution (Amores et al.,^1998^; Postlethwait et al.,^1998^) generated two paralogous copies for many of the genes typically used as markers of neuronal differentiation in vertebrates. The more than 400 million years of evolution since the genome duplication have changed the nucleotide sequences of most paralogous gene pairs sufficiently such that antisense probes might not cross-react. The expression patterns of such duplicated genes often differ, and they are not exactly expressed in the same subset of cells but very often in the same region with overlapping expression patterns. Paralogous genes were preserved during evolution, because in many cases their original complex functions and expression patterns were subject to subfunctionalization, such that each paralog took over some functions of the ancestral single gene (Force et al.,^1999^). In some cases, novel subfunctions might have emerged.

Regarding the catecholaminergic systems, previous studies in zebrafish have focused on using one marker, *tyrosine hydroxylase*, to identify DA and NA neurons. However, it was recently demonstrated that two paralogous *th* genes exist in teleosts, *th1* and *th2* (Candy and Collet,^2005^). Here, we investigated the expression pattern of the duplicated nonallelic genes *th1* and *th2* in the juvenile zebrafish brain. We chose juvenile brains of 30-day-old zebrafish to enable us to perform gene expression analysis in whole-mount preparations rather than on serial sections. From our point of view, analysis of the whole brain has the important advantage of allowing us to relate the expression better to the three-dimensional anatomical framework of the brain. Juvenile brains are still small enough to allow WISH and immunohistochemistry with optimized protocols, and at the same time their anatomy is largely identical to that of the adult zebrafish brain (Wullimann et al.,^1996^).

Our analysis revealed that, whereas *th1* is expressed in the DA neurons of the tel- and diencephalic DA clusters and the NA neurons of the locus coeruleus and the medulla oblongata in the hindbrain, *th2* is expressed exclusively in the diencephalon. We identified four *th2*-expressing neuronal groups that likely use dopamine as transmitter, including neurons of the posterior preoptic nucleus, the paraventricular organ, and two clusters in the caudal zone of periventricular hypothalamus.

The most anterior *th2*-expressing group was located in the anterior periventricular hypothalamus, in a region of the preoptic area that, by comparison with the zebrafish brain atlas (Wullimann et al.,^1996^), potentially correlates with the magnocellular (PM) and/or parvocellular preoptic nuclei. Insofar as we observed that this *th2*-expressing group is also TH1-ir, we believe that it has already been included in the description of zebrafish CA distribution in previous studies (Kaslin and Panula,^2001^; Ma,^2003^). However, although Ma (2003) reported TH immunoreactivity in the PM, Kaslin and Panula (2001) did not. Given that neurons of the PM are molecularly identifiable by the expression of nonapeptides, such as oxytocin/isotocin and vasopressin/vasotocin, and given that colocalization data for *th1/2* with such nonapeptides are still lacking in zebrafish, we decided to identify the location of this *th2*-expressing group with the more general term of *periventricular preoptic nucleus*. It must be considered, though, that coexpression of TH with oxytocin and vasopressin has been reported in neurons of the paraventricular and supraoptic nuclei in humans (Panayotacopoulou et al.,^1994^, ^2000^). Hence, the presence of *th1*/2-expressing cells in the zebrafish PM cannot be excluded until double-labeling experiments clarify this issue.

A second *th2*-expressing group was located in the intermediate hypothalamus, in a nucleus named by Rink and Wullimann (2001) the *paraventricular organ*, a term that has also been used to identify additional hypothalamic nuclei lining the third ventricle (Kaslin and Panula,^2001^).

Although we could show that *th1* and *th2* expression patterns overlap extensively in the anterior hypothalamus, two major populations of cerebrospinal fluid (CSF)-contacting neurons in the nuclei of the lateral and posterior recesses almost exclusively express *th2*, whereas the *th1* contribution is only minor in these areas. Interestingly, two populations of cells in the caudal hypothalamus have been previously described as strongly DA-ir but not or scarcely TH-ir in other teleosts (Batten et al.,^1993^; Ekström et al.,^1990^, ^1992^; Meek and Joosten,^1993^; Meek et al.,^1989^; Sas et al.,^1990^; Yoshida et al.,^1983^). To explain this apparent discrepancy, the CSF-contacting neurons of the caudal periventricular hypothalamus have been suggested to accumulate DA by a mechanism of uptake from the ventricular CSF rather than synthesizing it (Ekström et al.,^1990^; Smeets and González,^1990^). Although such a mechanism may actually occur, our data rather suggest that the neurons of the lateral and posterior recesses in the caudal hypothalamus do produce DA using TH2 as entry enzyme of CA biosynthesis instead of TH1.

Both *th1* and *th2* expression are absent from the midbrain, revealing that zebrafish do not form a mesencephalic dopaminergic system. We also included *dopamine beta hydroxylase*, *dopa decarboxylase*, and *dopamine transporter* in our analysis to show whether any of the *th2*-expressing cells might indeed generate dopamine and use it as a synaptic neurotransmitter. Our analysis revealed that none of the *th2* cells is in *dbh*-expressing territories, so they are not noradrenergic. In contrast, most or all *th2*-expressing neurons are in territories where *ddc* and *dat* expression is also detected, indicating that they may generate dopamine and use the reuptake carrier during dopaminergic synaptic transmission. However, the levels of *dat* expression vary significantly and are hardly detectable in the DA neurons of the periventricular preoptic nucleus. Thus it is also possible that some of the DA neurons do not use synaptic reuptake as a major mechanism to cease neurotransmission but may contribute DA neurosecretory activity. Our observation of relatively low levels of *dat* expression in many dopaminergic groups of the juvenile brain made us consider whether there are any correlations between properties of DA neuronal groups and level of *dat* expression. We noted that DA groups with far-ranging projections, including those of the posterior tuberculum and pretectum (Kaslin and Panula,^2001^; McLean and Fetcho,^2004^; Rink and Wullimann,2002a), contain higher levels of *dat* expression than locally projecting DA neurons of, e.g., olfactory bulb or caudal hypothalamus. However, whether this is a significant correlation must be investigated.

We also investigated *th2* expression at embryonic stages when *th1* expression domains start to be established. *th2* Is very weakly expressed in only a small number of cells in 1- and 2-day-old zebrafish embryos; however, the anatomical locations in the ventral diencephalon correlate with those that we observed in juvenile brains. The fact that *th2* is only weakly expressed during embryonic stages but shows a strong expression in the juvenile brain suggests that *th1* neurons may predominantly provide DA activity in the embryonic and early larval brain, whereas *th2* contributes more significantly to its role in neurotransmitter synthesis at postembryonic stages. Furthermore, we speculate that the control of physiology and body functions exerted by the prominent caudal hypothalamic DA groups might emerge only in postembryonic stages when larvae become independent of the yolk supply and depend on external energy sources. Some of these activities may be exerted by innervation of the pituitary (Fryer et al.,^1985^) or through modulation of cardiorespiratory activity, as shown in rat by Dillon and Waldrop (1992). Given the relatively high levels of *dat* expression in the *th2*-expressing caudal hypothalamic groups, it is most likely that these groups exert functionally significant dopaminergic synaptic transmission in the hypothalamus.

It is tempting to correlate zebrafish CA neurons with the CA groups defined for mammals (Hökfelt et al.,^1984^). However, one example, OTP-specified neurons of the A11 group in mammals and Otp-specified neurons of the posterior tuberculum and hypothalamus in zebrafish, has revealed that direct comparison of anatomical locations might not always be informative. OTP-dependent A11 neurons are located predominantly in the alar plate in mammals, whereas the zebrafish groups are at the alar–basal boundary and in the hypothalamus. With these caveats, some correlations can still be drawn based on distinct anatomical positions and related to previously published suggestions on evolutionary relationships of DA groups (Holzschuh et al.,^2001^; Kaslin and Panula,^2001^; Ma and Lopez,^2003^; McLean and Fetcho,^2004^; Rink and Wullimann,2002b; Smeets and González,^2000^; Smeets and Reiner,^1994^). The caudal NA groups A1–A2 correspond to medulla oblongata groups in zebrafish, and, among A3–A7 groups, only the noradrenergic LC neurons appear to have correlates in zebrafish. Groups directly corresponding to A8–A10 are absent from zebrafish. For the hypothalamic A12 group, likely correlates in zebrafish include the PVO and caudal hypothalamic groups. By location, A13 likely correlates with the ventral thalamic group (numbered group 1) in zebrafish. A14 and A15 are difficult to correlate to distinct groups in the preoptic area and hypothalamus in zebrafish; candidate regions include the PvPp area of *th* expression in zebrafish for A14 and PPa and SC for A15. Finally, A16 olfactory group and A17 retinal group have direct anatomical counterparts in zebrafish.

In summary, our observations are consistent with the hypothesis that the two paralogous *tyrosine hydroxylase* genes have subfunctionalized in fish and are differentially regulated at the transcriptional level (Candy and Collet,^2005^). Our analysis has for the first time elucidated the full complement of dopaminergic and noradrenergic neuronal groups at juvenile stages of the zebrafish, when the brain anatomy is already very similar to that of adult and mature brain. Significantly, the additional *th2* expression domains not overlapping with *th1* described in this work explains the contradictions previously observed in the distribution of DA- and TH-ir neurons in other teleosts and nonmammalian vertebrates. Moreover, our data exclude the presence of a mesencephalic dopaminergic system in zebrafish and point at a greater complexity of the ventral diencephalic dopaminergic systems compared with previous knowledge based on *th1* analysis alone.

## Abbreviations

ac: anterior commissure
Ce: cerebellum
CM: corpus mamillare
d: diencephalon
D: dorsal telencephalic area
DP: dorsal posterior thalamic nucleus
GC: griseum centrale
H: hypothalamus
Ha: habenula
hac: habenular commissure
Hc: caudal zone of periventricular hypothalamus
Hd: dorsal zone of periventricular hypothalamus
Hv: ventral zone of periventricular hypothalamus
IL: inferior lobe of hypothalamus
LC: locus coeruleus
LR: lateral recess of diencephalic ventricle
m: mesencephalon
MO: medulla oblongata
n: notochord
NIn: nucleus interpeduncularis
OB: olfactory bulb
OT: optic tract
PM: magnocellular preoptic nucleus
PO: preoptic region
poc: postoptic commissure
PPa: parvocellular preoptic nucleus, anterior part
PPp: parvocellular preoptic nucleus, posterior part
PR: posterior recess of diencephalic ventricle
Pr: pretectum
PT: posterior tuberculum
PTN: posterior tuberal nucleus
PVO: paraventricular organ
PvPp: posterior periventricular preoptic nucleus
r: rhombencephalon
Ret: retina
SC: suprachiasmatic nucleus
SP: subpallium
SR: superior raphe
t: telencephalon
TeO: optic tectum
TL: torus longitudinalis
V: ventral telencephalic area
VL: ventrolateral thalamic nucleus
VM: ventromedial thalamic nucleus
