# Supplementary figures and images for: Expression of the paralogous tyrosine hydroxylase encoding genes th1 and th2 reveals the full complement of dopaminergic and noradrenergic neurons in zebrafish larval and juvenile brain
Source: J Comp Neurol. 2009 Sep 16;518(4):423–38. doi: 10.1002/cne.22213 (PMC2841823; doi:10.1002/cne.22213)

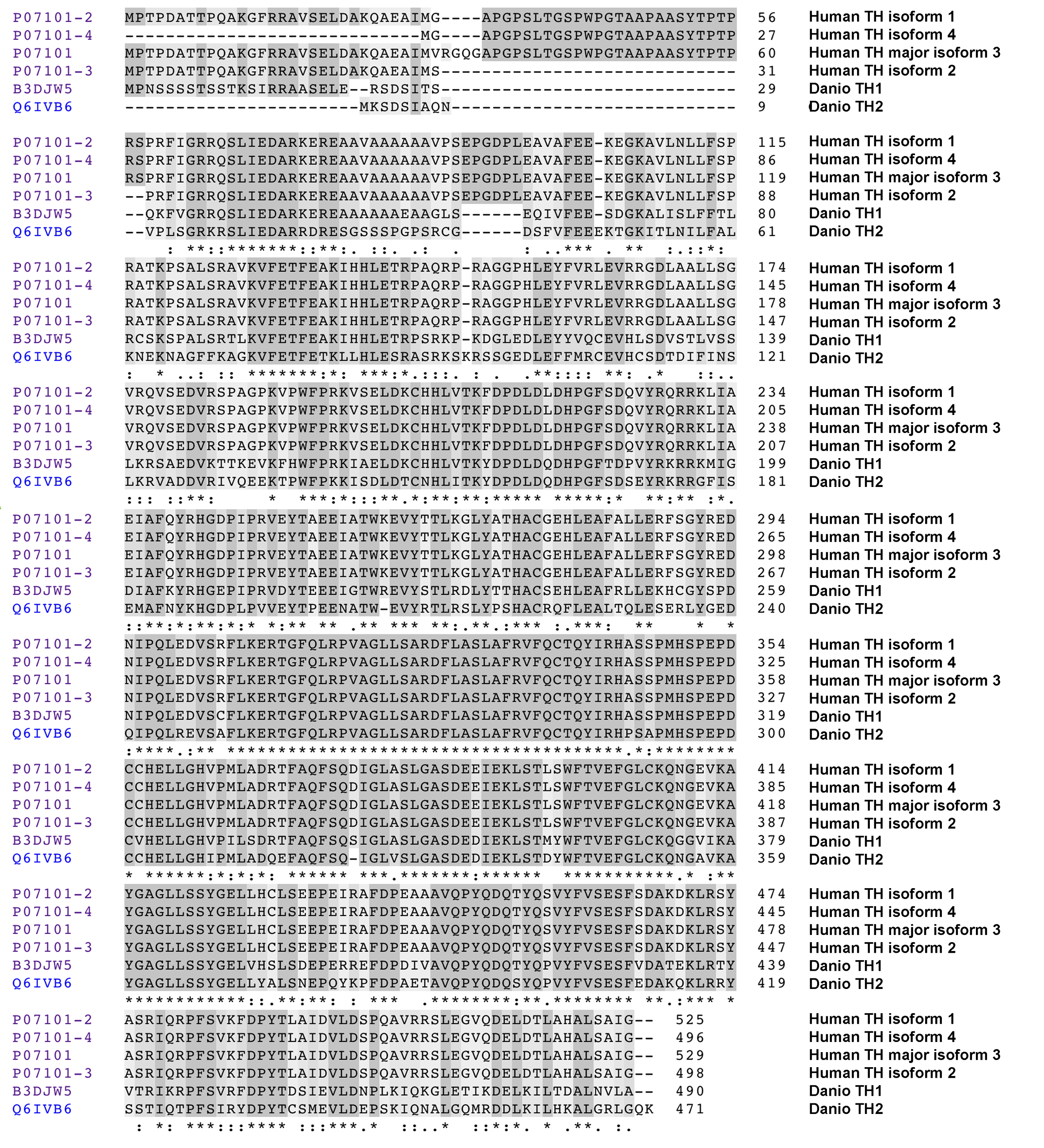

Supplement: Supplementary file 1 [file cne0518-0423-SD1.tif]

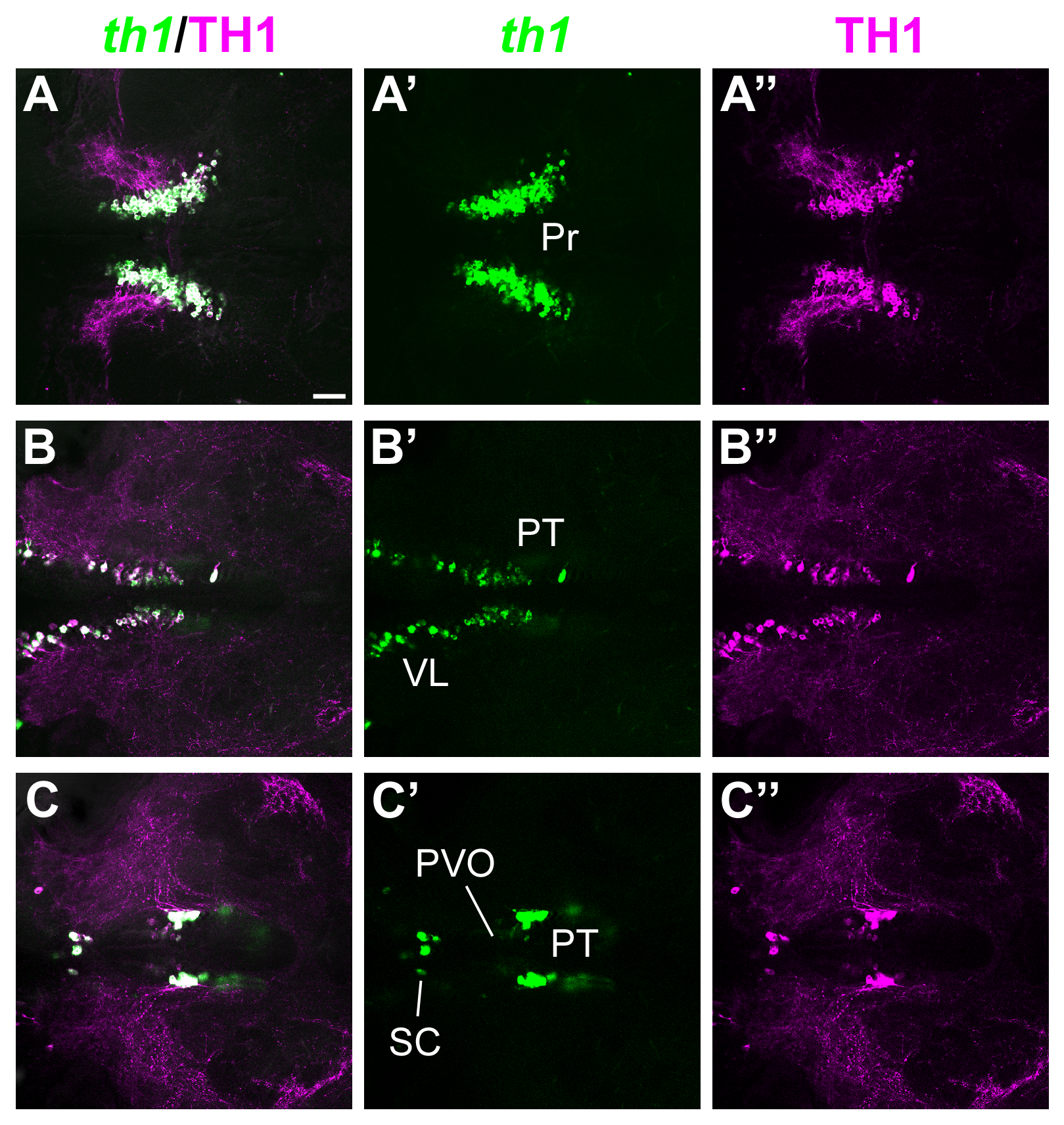

Supplement: Supplementary file 2 [file cne0518-0423-SD2.tif]

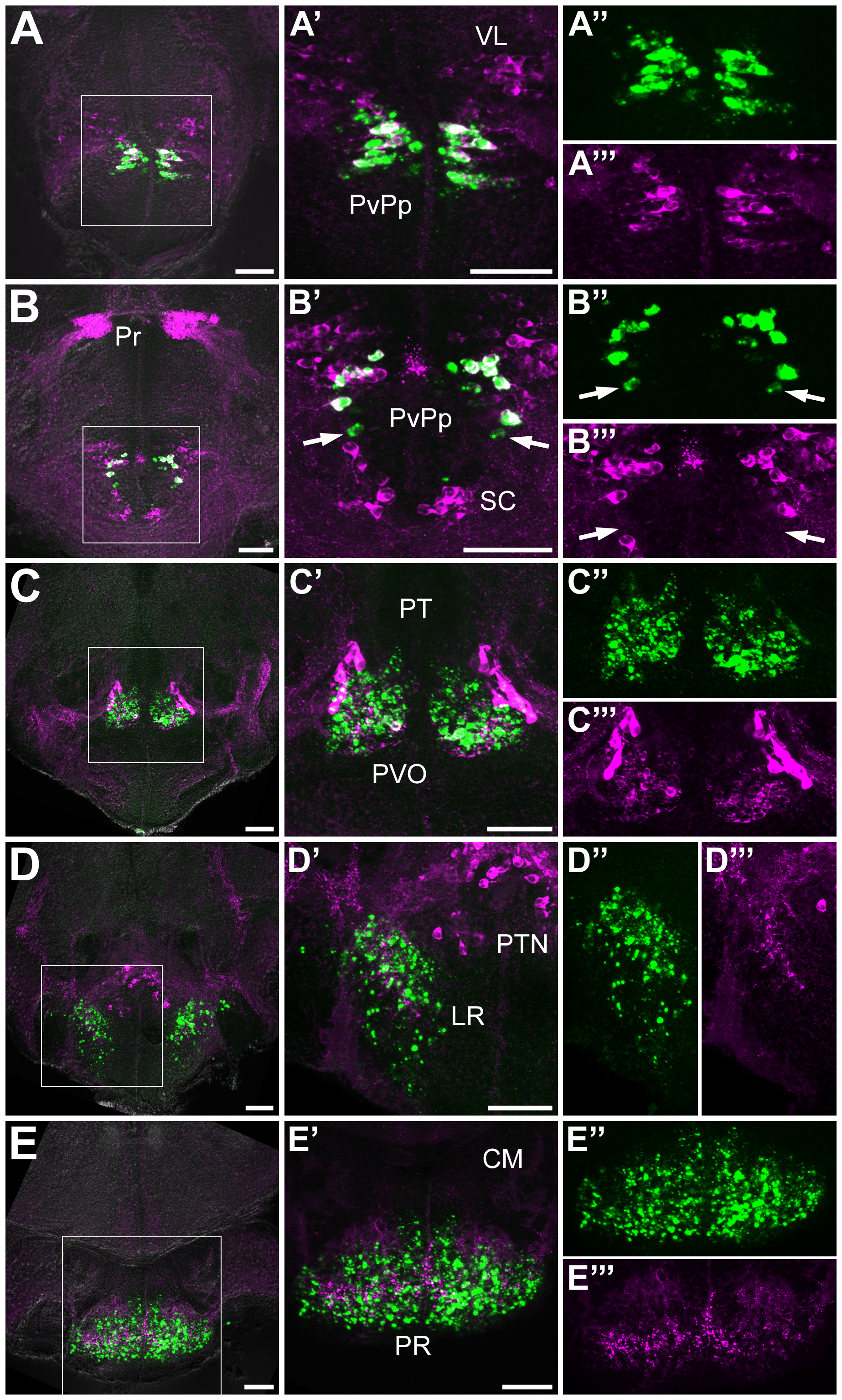

Supplement: Supplementary file 3 [file cne0518-0423-SD3.tif]
